# Determining RNA Natural Modifications and Nucleoside Analog-Labeled Sites by a Chemical/Enzyme-Induced Base Mutation Principle

**DOI:** 10.3390/molecules28041517

**Published:** 2023-02-04

**Authors:** Ziming Bao, Tengwei Li, Jianzhao Liu

**Affiliations:** 1 MOE Key Laboratory of Macromolecular Synthesis and Functionalization, Department of Polymer Science and Engineering, Zhejiang University, Hangzhou 310058, China; 2 Life Sciences Institute, Zhejiang University, Hangzhou 310058, China

**Keywords:** RNA modifications, nucleoside analogs, chemical sequencing of RNA modifications, chemical/enzyme-induced reverse transcription misincorporation, RNA metabolic labeling

## Abstract

The natural chemical modifications of messenger RNA (mRNA) in living organisms have shown essential roles in both physiology and pathology. The mapping of mRNA modifications is critical for interpreting their biological functions. In another dimension, the synthesized nucleoside analogs can enable chemical labeling of cellular mRNA through a metabolic pathway, which facilitates the study of RNA dynamics in a pulse-chase manner. In this regard, the sequencing tools for mapping both natural modifications and nucleoside tags on mRNA at single base resolution are highly necessary. In this work, we review the progress of chemical sequencing technology for determining both a variety of naturally occurring base modifications mainly on mRNA and a few on transfer RNA and metabolically incorporated artificial base analogs on mRNA, and further discuss the problems and prospects in the field.

## 1. Introduction

**Figure 1:**
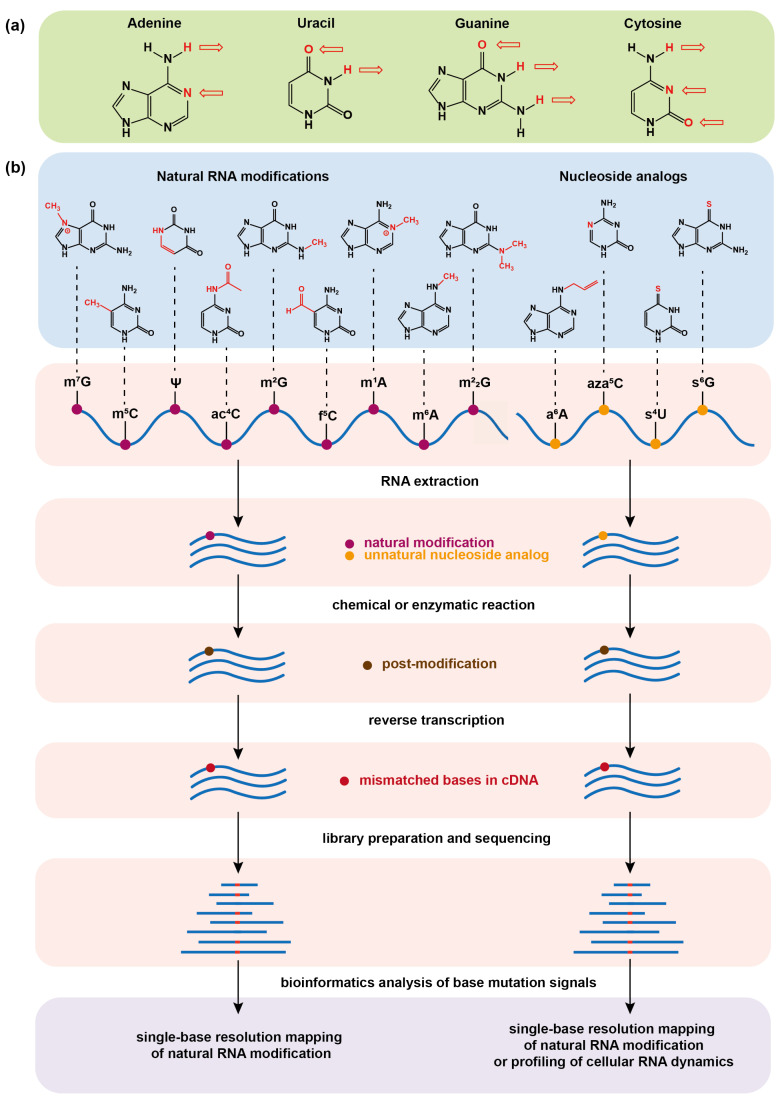
(**a**) Hydrogen bonding of the four basic bases that make up RNA in the Watson–Crick base-pairing principle. The arrow symbolizes the hydrogen bond and its direction points to the receptor of the hydrogen bond. (**b**) Schematic illustrations of chemical sequencing methods for determining natural RNA modifications at single base resolution or profiling cellular RNA dynamics by metabolic labeling. The chemical or enzymatic reactions convert natural RNA modifications or metabolically incorporated nucleoside analogs into new structural variants (post-modifications), which can induce base mismatch during reverse transcription of RNA into complementary DNA (cDNA) and can eventually be read by base mutation signals in the sequencing data.

RNA is an essential biomolecule inside cells and not only serves as the carrier of genetic information but also plays regulatory functions. With the development of chromatography technology, it has been discovered that RNA contains a great variety of chemically modified bases other than the four canonical nucleobases adenine (A), cytosine (C), guanine (G), and uracil (U). Since the discovery of pseudouridine in 1951, more than 170 kinds of RNA modifications have been identified [1]. Typically, the messenger RNA (mRNA) modifications, such as $N^{6}$-methyladenosine (m${}^{6}$A) [2], 5-methylcytidine (m${}^{5}$C) [3,4], $N^{7}$-methylguanosine(m${}^{7}$G) [5,6], $N^{4}$-acetylcytidine (ac${}^{4}$C) [7,8], $N^{5}$-formylcytidine (f${}^{5}$C) [9], pseudouridine (Ψ) [10,11], and 2${}^{\prime }$-*O*-methylation (Nm) [12,13], and transfer RNA (tRNA) modifications $N^{1}$-methyladenosine (m${}^{1}$A) [14,15], $N^{2}$-methylguanosine (m${}^{2}$G), and $N^{2}$,$N^{2}$-dimethylguanosine (m${}^{2}{}_{2}$G) [16] are among the research frontiers [17,18]. These modifications affect the flow of genetic information and bring a crucial layer of regulation to gene expression [19,20,21]. However, most of the modifications still follow the Watson–Crick base-pairing principle (Figure 1a) leading to the inability of widely used Next Generation Sequencing (NGS) technologies to distinguish them from common bases, which causes great difficulty in mapping modifications and exploring their biological functions. The low abundance of some modifications also brings great challenges to researches on rare samples.

With the unremitting efforts of researchers, a number of sequencing methods have been developed over the past decade in order to map different modifications in a transcriptome-wide manner and other RNAs [22,23,24,25]. These advances have greatly facilitated the discoveries of the functions of RNA modifications. For example, the most abundant modification m${}^{6}$A on mRNA has been found to affect RNA transcription, splicing, stability, translation, as well as immune regulations [26,27,28,29,30,31,32,33,34]. Among these sequencing methods, chemical sequencing tools have attracted considerable attention because they take advantages of chemical/enzymatic treatment of RNA modification sites to alter their base-pairing features and then induce base misincorporation during reverse transcription (RT), and eventually detect the modification sites on the basis of nucleobase mutation signatures from the NGS (Figure 1b). Chemical sequencing has the advantages of single-base resolution, low false positive in detection, and simple experimental protocol, and it has been widely used to identify natural mRNA modifications and map them at a transcriptome-wide scale.

In addition to natural RNA modifications, artificial nucleoside analogs [35,36] have been used to metabolically label cellular nascent RNAs as tags to study RNA dynamics (Figure 1b) [37,38]. The RNA dynamic is closely related to the processes of RNA synthesis and degradation. Traditional RNA sequencing (RNA-seq) generally reports the steady-state level of cellular RNA [39], offering the information about the relative abundance of each transcript in the entire transcriptome. In order to capture temporal RNA dynamics, nucleoside analogs with both biocompatibility for metabolic labeling of RNA and capability for chemical sequencing are highly useful, because they can be incorporated into nascent RNA in a pulse manner and can then be quantified by chemical-induced base mutation signals of the labeled RNA in RNA-seq at a certain chasing time point. 4-thiouridine (4SU), 6-thioguanosine (6SG), and $N^{6}$-allyladenosine (a${}^{6}$A) are the reported nucleoside analogs for metabolic labeling of RNA, and the corresponding strategies for their chemical sequencing have been developed [40,41,42,43], which enables a transcriptome-wide study of RNA metabolism in a variety of key biological systems. Furthermore, the metabolic labeling strategy has been introduced to post-transcriptional RNA modification systems, and is thus gradually evolving into a powerful tool for identifying natural RNA modifications [44,45].

In this review, we summarize the recent progress of chemical sequencing technologies for determining both a variety of naturally occurring base modifications and metabolically incorporated artificial base analogs on mRNA (Figure 1b). The chemical sequencing tools mainly focus on specifically converting the modified bases or base analogs on RNA into new structural variants by chemical or enzymatic reactions, and detect them in a principle of base mismatch during RNA RT into complementary DNA (cDNA). We also discuss the problems and prospects in this field.

## 2. Chemical Sequencing Methods for Detecting RNA Natural Modifications

**Figure 2:**
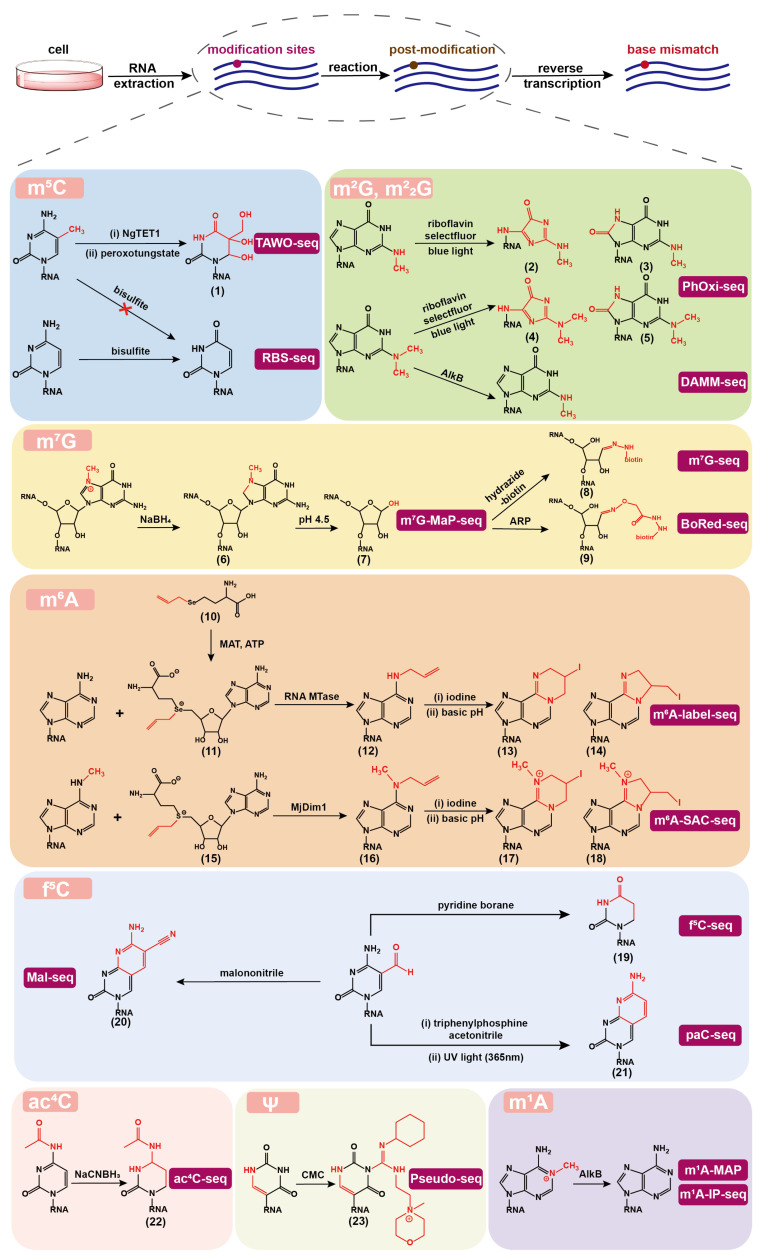
Various chemical or enzymatic reactions for converting natural RNA modifications into post-modified forms, which can induce base mismatch during reverse transcription of RNA into cDNA and thus can be identified at single base resolution in the corresponding high-throughput sequencing methods. TAWO-seq: TET-assisted peroxotungstate oxidation sequencing; RBS-seq: RNA bisulfite sequencing; PhOxi-seq: photo-oxidative sequencing; DAMM-seq: demethylation-assisted multiple methylation sequencing; m${}^{7}$G-MaP-seq: m${}^{7}$G mutational profiling sequencing; BoRed-seq: borohydride reduction sequencing; m${}^{6}$A-SAC-seq: m${}^{6}$A-selective allyl chemical labeling and sequencing; Mal-seq: selective malononitrile-mediated labeling and sequencing; NgTET1: Naeglaria Tet-like oxygenase; AlkB: alpha-ketoglutarate-dependent dioxygenase; ARP: *N*-(aminooxyacetyl)-*n*′-(d-biotinoyl) hydrazine; MAT: methionine adenosyltransferase; ATP: adenosine triphosphate; RNA MTase: RNA methyltransferase; MjDim1: Methanocaldococcus jannaschii homolog; CMC: *N*-cyclohexyl-*N*′-(2-morpholinoethyl) carbodiimide metho-*p*-toluenesulphonate.

In this section, we summarize the chemical sequencing methods that identify RNA natural modifications with the aid of chemical/enzymatic post-treatment (Figure 2). In general, they consist of the following steps. First, cellular RNA is extracted; second, specific chemical or enzymatic reactions are performed to alter the base-pairing properties; third, appropriate reverse transcriptase is applied to read through the RNA post modification sites, which introduce the mismatches during the synthesis of cDNA; last, cDNA libraries are constructed and sequenced to determine modification sites on the basis of mutation signatures from bioinformatics analysis.

### 2.1. m${}^{5}$C Detection

m${}^{5}$C is present in both DNA (commonly named 5mC) and RNA and has been intensively studied [46]. In 1992, bisulfite sequencing (BS-seq) was developed to detect cytidine methylation on a genome-wide scale [47]. Under an acidic pH and bisulfite treatment, the C deaminates and generates uridine sulfonate, which then desulfonates to uridine under a basic pH. As 5mC modifications are inert to this reaction, it is possible to characterize genome-wide 5mC by identifying non-converted C’s in the sequencing data. Due to its broad application and base resolution, BS-seq is the gold standard for detecting 5mC across the entire genome. However, it is unable to differentiate between 5mC and 5-hydroxymethylcytidine (named 5hmC in DNA and hm${}^{5}$C in RNA), and bisulfite treatment degrades DNA, limiting its application to rare and valuable samples.

BS-seq has also been extended to detect RNA m${}^{5}$C sites in various species [48,49,50]. However, bisulfite reactions on RNA are severely hindered by their complex secondary structures, resulting in the incomplete conversion of C-to-U and unsatisfactory results. In 2019, Khoddami et al. developed RNA bisulfite sequencing (RBS-seq) [51], a method to sensitively detect m${}^{5}$C, Ψ and m${}^{1}$A modifications transcriptome-wide at single-base resolution. By mixing RNAs with formamide and heating, the robustness of m${}^{5}$C detection was improved, and the interference induced by RNA secondary structures was eliminated to a large extent. A total of 486 m${}^{5}$C sites in human HeLa cell mRNA were detected, showing a much smaller number than the previously reported thousands of sites [52].

Additionally, compared with DNA, RNA is more vulnerable to high temperatures and alkaline conditions that are required for bisulfite treatment and affect the quality of sequencing reads. In order to protect RNA from degradation, shortening the reaction time decreases the deamination efficiency and eventually leads to a false positive effect. These problems have not yet been well resolved. In this regard, RBS-seq is more suitable for detecting substrates with a high abundance of m${}^{5}$C modification. As for the drawbacks of not being powerful enough to distinguish between m${}^{5}$C and hm${}^{5}$C [53], appropriate methods have been developed and successfully applied. Initially, the peroxotungstate oxidation sequencing (WO-seq) was developed to detect hm${}^{5}$C [54]. WO-seq employs peroxotungstate to oxidize hm${}^{5}$C to trihydroxylated-thymine (*^th^*T, **1**), which is then distinguished as T by a thermostable group II intron reverse transcriptase (TGIRT) during cDNA synthesis. Subsequently, WO-seq was upgraded to the TET-Assisted WO-Seq (TAWO-seq) (Figure 2), which labels natural hm${}^{5}$C by β-glucosyltransferase (β-GT) first to protect it from conversion to *^th^*T and further converts m${}^{5}$C into hm${}^{5}$C by the Naeglaria Tet-like oxygenase (NgTET1) [55]. These newly generated hm${}^{5}$C sites, equivalent to m${}^{5}$C sites, can be detected by WO-seq. This technique is anticipated to have a lower rate of false positives because it does not result in mutations on a large number of C sites.

### 2.2. m${}^{2}$G and m${}^{2}{}_{2}$G Detection

m${}^{2}$G and m${}^{2}{}_{2}$G are prevalent modifications on cellular RNAs. m${}^{2}$G has almost no impact on the Watson–Crick base pairing principle, while m${}^{2}{}_{2}$G, like m${}^{1}$A, $N^{1}$-methylguanosine (m${}^{1}$G), and $N^{3}$-methylcytidine (m${}^{3}$C), is a modification that does not conform to the base-pairing principle and causes truncation or mutation during RT. It should be mentioned that m${}^{2}{}_{2}$G together with m${}^{1}$A, m${}^{1}$G, and m${}^{3}$C are highly enriched in tRNA, making tRNA hard to be profiled by the conventional sequencing techniques.

Chung et al. was inspired by the examples of visible light photoredox chemistry on RNA and DNA, and developed photo-oxidative sequencing (PhOxi-seq) [56]. Under the irradiation of blue light, Riboflavin and selectfluor (1-Chloromethyl-4-fluoro-1,4-diazoniabicyclo[2.2.2]octane bis(tetrafluoroborate)) were used as a photocatalyst and an oxidant, respectively, to convert m${}^{2}$G and m${}^{2}{}_{2}$G to potential products (**2**–**5**) (Figure 2), which pair randomly during RT and generate mutation signals in cDNA sequencing. The results showed that the mutation signal was generated at the m${}^{2}$G sites, and the read through rate at the m${}^{2}{}_{2}$G sites increased 6-fold. However, the m${}^{1}$G sites also produce mutations, which lead to ineffectively distinguishing m${}^{1}$G from m${}^{2}$G when this method is used alone.

As for m${}^{2}{}_{2}$G detection, three methods named AlkB-facilitated RNA methylation sequencing (ARM-seq) [57], demethylase-thermostable group II intron RT tRNA sequencing (DM-tRNA-seq) [58], and demethylation-assisted multiple methylation sequencing (DAMM-seq) [59] were developed. ARM-seq treated RNA samples with AlkB enzymes to remove the m${}^{1}$A, m${}^{1}$G, and m${}^{3}$C sites before sequencing and finally increased the abundance and diversity of reads when analyzing tRNA modifications in Saccharomyces cerevisiae and human cell lines. Since wild-type AlkB showed poor activity in removing m${}^{1}$G, DM-tRNA-seq was performed using a mixture of the wild-type and the D135S AlkB mutant and also used TGIRT in RT to get a higher read-through rate. The results demonstrated that more than 80% of m${}^{1}$A, m${}^{3}$C and about 70% of m${}^{1}$G modifications could be removed, while m${}^{2}{}_{2}$G modifications, which were present in about 20% of the tRNA, could not be effectively removed. Similarly, DAMM-seq combines human immunodeficiency virus (HIV) reverse transcriptase and AlkB D135S demethylase to simultaneously detect multiple modifications including m${}^{2}{}_{2}$G. To alleviate the issue that m${}^{2}{}_{2}$G is not effectively demethylated, Dai et al. [60] screened demethylases with a focus on m${}^{2}{}_{2}$G. They found that the AlkB D135S/L118V mutant selectively converts the m${}^{2}{}_{2}$G to m${}^{2}$G which will then improve the efficiency of tRNA sequencing. Wang et al. [61] also developed a high-throughput sequencing method for screening AlkB demethylation activity on RNA and DNA substrates, which committed to developing other functional AlkB enzyme variants in the future. Single-base resolution identification method can be developed with the help of demethylases that can specifically target m${}^{2}{}_{2}$G.

### 2.3. m${}^{7}$G Detection

m${}^{7}$G is well-known to exist in the 5′ cap of eukaryotic mRNA, as well as in the internal regions on tRNA and rRNA [62,63,64]. It has been revealed to play a crucial role in the mRNA maturation, nuclear export, stability, and translation initiation [65]. For example, m${}^{7}$G sites in mRNA cap act as a unique molecular module that recruits cellular proteins and mediates cap-associated biological functions. Even though the methylation significantly alters the charge density of RNA, m${}^{7}$G does not interfere with the base pairing during the RT process.

With the tremendous efforts of researchers, three chemical sequencing methods for m${}^{7}$G were developed in 2019. Zhang et al. developed the m${}^{7}$G-seq [5], which took advantage of the unique chemical reactivity of m${}^{7}$G in the reduction-induced depurination reaction. The nitrogen atom attached to the methyl group on the five-membered ring of the m${}^{7}$G is positively charged. m${}^{7}$G is particularly sensitive to NaBH${}_{4}$-mediated reduction and can undergo an addition reaction on the double bond in the five-membered ring (**6**), while the unmodified G does not undergo this reaction. Further, an acidic condition can induce the depurination of reduced m${}^{7}$G and lead to the formation of an abasic site (**7**), which can be conjugated to the biotin-modified hydrazide (**8**) for pull-down enrichment. The biotinylated sites would generate base misincorporation during RT using HIV reverse transcriptase. m${}^{7}$G-seq has successfully mapped m${}^{7}$G sites in human mRNA, rRNA, and tRNA at single nucleotide resolution. Enroth et al. developed m${}^{7}$G-MaP-seq [6], which differs from m${}^{7}$G-seq in that instead of capturing and enriching the abasic site, RT was directly performed on the abasic site using Primescript MMLV reverse transcriptase in order to acquire the base mismatch signals. The results are composed of insertions, deletions, and all possible types of mutations, leading to the difficulty in bioinformatic analysis. The Borohydride Reduction sequencing (BoRed-seq) [66] developed by Pandolfini et al. is similar to m${}^{7}$G-seq in some ways, with the main difference being the conjugation reaction using *N*-(aminooxyacetyl)-*n*′-(D-biotinoyl) hydrazine (ARP) to introduce biotin group (**9**). Future efforts are needed in enhancing the reaction’s efficiency to generate stable conjugates that induce mutations.

### 2.4. m${}^{6}$A Detection

m${}^{6}$A is the most abundant internal modification of mRNA and the first mRNA modification mapped. The growing evidences suggest that m${}^{6}$A determines mRNA fate and plays an essential regulatory role in physiological and pathological processes [67,68]. In order to study m${}^{6}$A functions, precise detection of m${}^{6}$A is highly necessary. A variety of high-throughput sequencing methods for m${}^{6}$A detection have been developed in the past decade, and have greatly advanced the study of m${}^{6}$A functions. m${}^{6}$A-seq [69] or named MeRIP-seq (methylated RNA immunoprecipitation followed by NGS) [70] is the first high-throughput sequencing method reported in 2012, and it relies on m${}^{6}$A antibody to enrich m${}^{6}$A-containing RNA followed by NGS. To date, the majority of the published studies have used this technique. In order to address the issue of the specificity of m${}^{6}$A antibody, Ye et al. developed m${}^{6}$A-SEAL-seq (m${}^{6}$A selective chemical labeling method), an antibody-free method through a dithiothreitol (DTT)-mediated thiol-addition chemical reaction [71]. This method has lower non-specificity and higher positive rate and can also be used for specific identification of cap m${}^{6}$Am. However, the disadvantage of a resolution of only 100 to 200 nt limits the applications for high-resolution mapping.

*S*-adenosyl-L-methionine (SAM) is a cofactor for virtually all known RNA and DNA methyltransferases (MTases), which transfer the methyl group of SAM to the specific methylation sites [72]. Various SAM analogs have been synthesized and used to study the catalytic activity of MTases and identify new MTase substrates for more than two decades [73]. However, SAM derivatives are less stable under the ambient reaction conditions and are hard to be internalized through the cell membranes due to its nature with charge [74]. These disadvantages limit the direct applications of SAM derivatives in cellular metabolic labeling. In a living cell, SAM is usually generated from adenosine triphosphate (ATP) and L-methionine in the presence of methionine adenosyltransferase [75]. In this regard, the application of L-methionine derivatives, which are precursors of SAM’s, for metabolic labeling is a feasible approach to mark and identify the nucleic acid modification sites. In 2018, Hartstock et al. synthesized *Se*-propargyl-L-selenohomocysteine to enable the metabolic labeling of RNA nucleosides with a propargyl group and enriched these labeled RNAs by click conjugation chemistry for further RNA sequencing [44]. In 2020, Shu et al. developed m${}^{6}$A-label-seq [45], which uses *Se*-allyl-L-selenohomocysteine (**10**) for metabolic labeling of mRNA (Figure 2). The methyl group on the enzyme cofactor SAM is replaced with an allyl group to generate allyl-*Se*-adenosyl-L-methionine (**11**), leading to a${}^{6}$A modification (**12**) via a methylation metabolic pathway at sites that would otherwise be m${}^{6}$A. The orthogonal efficient iodine-induced cyclization reaction generates major product (**13**,**14**), which results in base mismatches during RT, allowing the detection of m${}^{6}$A as an A to C/T/G mutation in RNA sequencing [42]. This method has been utilized to successfully mapped m${}^{6}$A modifications in mRNAs from human HeLa, HEK293T and mouse H2.35 cell lines. An orthogonal m${}^{6}$A detection assay has been performed to validate the mapped sites. It should be noted that this method has the disadvantage of a low labeling rate, and further MTase protein engineering is needed to easily the accommodate allyl group.

Inspired by the above work, the recently developed m${}^{6}$A-selective allyl chemical labeling and sequencing (m${}^{6}$A-SAC-seq) [76] utilizes the Methanocaldococcus jannaschii homolog MjDim1 to specifically modify m${}^{6}$A in the presence of allyl-*S*-adenosyl-L-methionine (**15**) to convert original m${}^{6}$A sites into $N^{6}$-allyl,$N^{6}$-methyladenosine (a${}^{6}$m${}^{6}$A) sites (**16**). a${}^{6}$m${}^{6}$A can be converted to cyclized forms as homologs of 1,$N^{6}$-ethanoadenine (**17**,**18**) via iodine-induced cyclization reaction, and these products result in base misincorporation upon RT using HIV reverse transcriptase. With this rationale, m${}^{6}$A sites on transcriptome-wide mRNAs from HeLa, HEK293 and HepG2 cell lines have been mapped at base resolution. It should be mentioned that m${}^{6}$A-SAC-seq is limited in detecting less frequently m${}^{6}$A-methylated Am${}^{6}$AC motif, while the detection of m${}^{6}$A sites with high stoichiometry in this motif is not affected. It is revealed that stoichiometric information of over 70% of DRACH sequences is obtained. This method can start with a low input of 30 ng and can also be used to study m${}^{6}$A dynamics during cell differentiation.

### 2.5. f${}^{5}$C Detection

When Liu and colleagues developed a technology to identify 5mC and 5hmC sites in DNA [77], they discovered that f${}^{5}$C (named 5fC in DNA) and $N^{5}$-carboxycytidine (named 5caC in DNA and ca${}^{5}$C in RNA) could be transformed into dihydrouridine (DHU) (**19**) with pyridine borane. Wang et al. applied this idea to detect f${}^{5}$C in transcriptomic RNAs and developed the f${}^{5}$C-seq [78]. The f${}^{5}$C is converted into DHU by pyridine borane, and DHU is paired with A during RT to induce a C to T mutation in the sequencing result. The single-base resolution f${}^{5}$C map in *S. cerevisiae* mRNAs shows that f${}^{5}$C is a widespread mRNA modification and is more likely present at the third position of the codon. Similarly, the abovementioned bisulfite sequencing and borohydride sequencing methods can also be used to detect f${}^{5}$C sites [79,80]. Some putative f${}^{5}$C sites uncovered by these three methods may arise from $N^{5}$-carboxylcytidine (ca${}^{5}$C) or other modifications in RNA, leading to false positive signals. Therefore, it is necessary to develop more accurate assays to detect f${}^{5}$C in RNA.

Inspired by the DNA 5fC detection rationale [81], Li et al. developed an RNA f${}^{5}$C sequencing method based on the malononitrile-induced C-to-T mutations, named Mal-seq [82]. This method treats f${}^{5}$C with malononitrile and the adduct (**20**) induces base incorporation by reverse transcriptase. Importantly, the levels of related modifications in total RNA, such as m${}^{5}$C and hm${}^{5}$C, remain unchanged, indicating that the malononitrile-mediated transformation is specific to f${}^{5}$C and mild enough. With this method, mt-tRNA(Met) in human HEK293T cells has been characterized to be fully modified with f${}^{5}$C. f${}^{5}$C shows high abundance in mammals but is absent in lower eukaryotes.

Another recently developed paC-seq [83] takes advantage of the electrophilicity of the C5-carbonyl group in f${}^{5}$C and photo-assisted $N^{4}$,$C^{5}$-cyclization reaction to specifically generate f${}^{5}$C adducts, which can lead to base misincorporation in the subsequent RT process. By photochemically labeling the f${}^{5}$C sites under UV light (365 nm) in the presence of the Vittich reagent triphenylphosphine acetonitrile, the cytidine analog 4,5-pyridin-2-amine-cytidine is formed and named paC (**21**). Out of the oligonucleotides tested, 70.2–70.9% show RT-induced mismatches, 98% of which are C to U mutations. Meanwhile, paC is an excellent fluorophore with a high quantum yield, making it possible to accurately determine f${}^{5}$C levels with a detection limit of 0.58 nM. This method is sensitive, robust, and antibody-free; however, it has not been utilized in cell and tissue studies.

### 2.6. ac${}^{4}$C Detection

ac${}^{4}$C is another modification that has been traditionally thought of as a rRNA and tRNA modification but has been recently suggested to be present in the mRNA [84]. The electron-deficient nature of ac${}^{4}$C pyrimidines makes itself prone to reduction, resulting in the reduced form $N^{4}$-acetyl-3,4,5,6-tetrahydrocytidine (**22**) (Figure 2). Thomas et al. [85] found that the reduced product can affect RT and cause base mismatch or truncations. The truncation occurs predominantly at the −1, 0, and +1 positions relative to the ac${}^{4}$C site. The RT enzyme TGIRT had the highest read-through efficiency after screening numerous reverse transcriptases and related reaction conditions. This technique has been applied to endogenous rRNA substrates, but it does not work on ac${}^{4}$C in densely modified targets such as tRNAs.

The ac${}^{4}$C-seq [86,87] offers some improvements to the previous method by using sodium cyanoborohydride (NaCNBH${}_{3}$) to react with the ac${}^{4}$C under acidic conditions. Faster kinetics and an increased base mutation rate have been observed. It has been proven sensitive enough to detect ac${}^{4}$C at a given site with as little as 4% stoichiometry. When hydrolyzed under a mild base condition prior to the reaction, the ac${}^{4}$C can be deacetylated to C. Therefore, deacetylated RNA can be chosen as a control to increase the signal-to-noise ratio. However, the result shows no conclusive evidence for the presence of ac${}^{4}$C in eukaryotic mRNAs. In contrast, the acRIP-seq [7], which uses antibodies to enrich the ac${}^{4}$C-containing RNA, suggests that the ac${}^{4}$C is present in eukaryotic mRNA. Therefore, the discrepancy remains.

### 2.7. Ψ Detection

Ψ is the most abundant nucleoside modification in non-coding RNA and enhances the function of tRNA and rRNA [88]. Several high-throughput sequencing methods have been developed to detect Ψ in RNA, including Ψ-seq [89], PSI-seq [90], Pseudo-seq [11], and CeU-seq [91]. These methods rely on the *N*-cyclohexyl-*N*′-b-(4-methylmorpholinium) ethylcarbodiimide (CMC) and its derivatives such as azide-modified CMC (N${}_{3}$-CMC) to react with pseudouridine to form the specific adducts [92]. More specifically, CMC can react with U, G, and Ψ residues to produce covalent products CMC-U, CMC-G and CMC-Ψ (**23**), respectively. The less stable CMC-U and CMC-G can be hydrolyzed upon base treatment, while CMC-Ψ is retained. Multiple reverse transcriptases, such as AMV and SuperScript III, produce a truncation at the CMC-Ψ site. Zhou et al. [93] optimized the conditions for RT and looked forward to detecting Ψ through the mutation principle. After testing various reverse transcriptases and evaluating the effects of different divalent cations, they found a higher read-through rate on the RNA oligonucleotide probe (over 80%) using SuperScript III reverse transcriptase in the presence of 3 mM Mn${}^{2+}$ or using HIV reverse transcriptase. When the same conditions were applied to the sample of human rRNA, a higher truncation rate and lower mutation rate than expected were observed. There is a vast room to develop new chemical/enzymatic reactions to specifically label Ψ, which enables the detection of Ψ through the base mutation principle.

### 2.8. m${}^{1}$A Detection

Due to its impaired base pairing, m${}^{1}$A can lead to mismatches or truncations during RT. m${}^{1}$A can be theoretically identified directly by performing high-throughput sequencing without chemical or enzymatic post-processing, however, the demethylation experiment of m${}^{1}$A or the conversion of m${}^{1}$A to m${}^{6}$A through Dimroth rearrangement reaction is widely used to eliminate background signals and increase the confidence of the map [94]. The reported m${}^{1}$A-ID-seq [14] demethylated group under the treatment of demethylase AlkB was used as the control. Eventually, 901 high-confident m${}^{1}$A peaks are identified in the transcriptome of HEK293T cells. The m${}^{1}$A-seq [15] takes advantages of Dimroth rearrangement m${}^{1}$A to m${}^{6}$A under alkaline conditions. By comparing the sequencing profiles before and after an alkaline treatment, the location of m${}^{1}$A sites are determined. The above two methods indicate that m${}^{1}$A is enriched in the 5${}^{\prime }$-untranslated region (5${}^{\prime }$-UTR) and the start codon, but Schwartz [95] thought that all 5${}^{\prime }$-UTR m${}^{1}$A sites are the result of incorrect bioinformatics analysis, which necessitates additional research.

Furthermore, m${}^{1}$A-MAP [96] was developed to improve the detection performance. Li et al. tested the read-through efficiency of several reverse transcriptases including AMV, SuperScript II, SuperScript III, and TGIRT under different conditions and found that TGIRT exhibited excellent read-through efficiency and relatively high mutation frequency at the m${}^{1}$A site. Based on the mutation analysis, m${}^{1}$A sites were found located in 5${}^{\prime }$-UTR of mRNA. Similarly, both m${}^{1}$A-Seq-TGIRT and m${}^{1}$A-seq-SS (SuperScript III) methods [97] utilized base mutation and truncation signals, respectively, to locate m${}^{1}$A. These methods mainly detected mature sites in rRNA and tRNA, but only 15 sites in mRNA and long-stranded non-coding RNA (lncRNA) were identified, 10 of which were located in cytosolic transcripts, and 5 of which were in mitochondria. Furthermore, Zhou [98] and colleagues developed a fluorescence-based directed evolution platform to evolve HIV reverse transcriptases that can both efficiently read through m${}^{1}$A and generate faithful mutation signatures. Two HIV variants, RT-733 and RT-1306, with the best read-through properties and mutation signatures at m${}^{1}$A sites, were developed. They applied them in m${}^{1}$A-IP-seq and discovered hundreds of new m${}^{1}$A sites in human mRNA, in addition to validating many of previously reported sites.

## 3. Chemical Sequencing Methods for Detecting Artificial Nucleoside Analogs Marked on RNA by Metabolic Labeling

**Figure 3:**
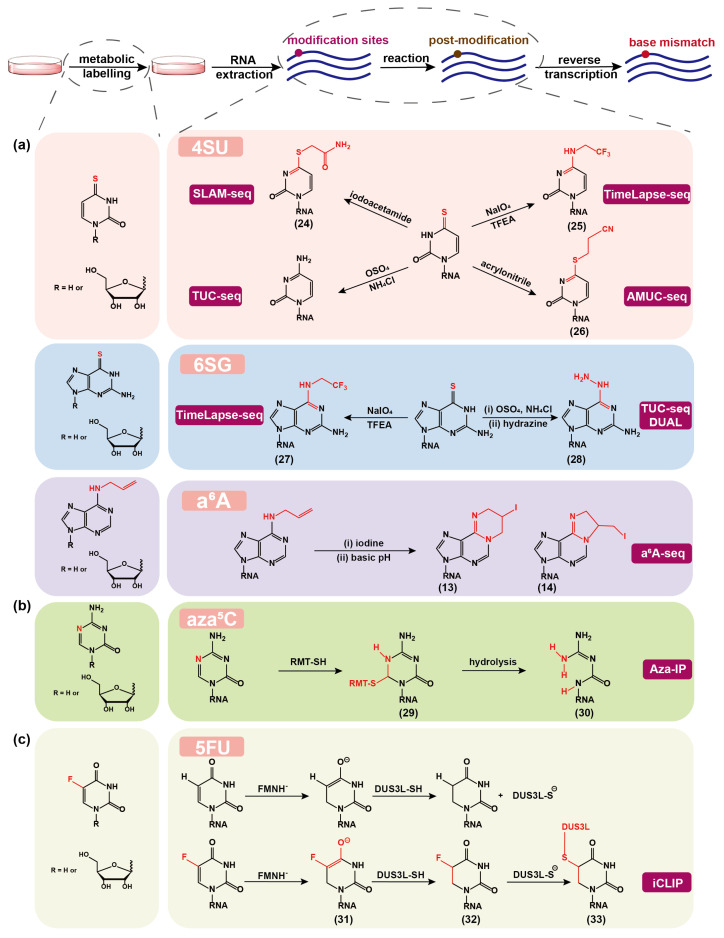
The nucleoside analogs and amino acid derivatives used for metabolic labeling of RNA and the associated chemical or enzymatic reactions for converting metabolically labeled nucleoside analogs into post-modified forms, which can mostly induce base mismatch during reverse transcription of RNA into cDNA and thus can be identified at single base resolution in the corresponding high-throughput sequencing methods. (**a**) The chemical rationales for profiling cellular RNA dynamics. (**b**) The chemical rationales for determining natural RNA modifications. (**c**) An enzymatic post-modification, which induces truncation instead of base mismatch during reverse transcription of RNA into cDNA, is shown as a comparison. SLAM-seq: thiol(SH)-linked alkylation metabolic sequencing; TUC-seq: thiouridine-to-cytidine conversion sequencing; AMUC-seq: acrylonitrile-mediated uridine-to-cytidine conversion sequencing; Aza-IP: 5-azacytidine–mediated RNA immunoprecipitation; iCLIP: individual-nucleotide resolution UV cross-linking and immunoprecipitation; TFEA: 2,2,2-trifluoroethylamine; RMT: m${}^{5}$C-RNA methyltransferases; FMN: flavin mononucleotide; DUS3L: a dihydrouridine synthase homolog.

In this section, we summarize the currently available chemical sequencing methods for detecting artificial nucleoside analogs that are marked on RNA through cellular metabolic labeling (Figure 3). Synthesized nucleoside analogs or the precursors of nucleoside-modifying enzyme cofactors can participate in the RNA synthesis and post-modification pathways, and thus RNA can be marked with artificial chemical tags, which can be determined by chemical sequencing methods site-specifically and quantitatively.

### 3.1. 4SU for RNA Labeling and Detection

4SU has become the most widely used uridine analog for studying RNA dynamics because of its great biocompatibility for metabolic labeling of RNA and its capability to specifically react with thiols and to be reversibly biotinylated for enrichment. 4SU is readily taken up by mammalian cells and converted to 4SUTP via the endogenous nucleotide salvage pathway [99]. For example, the major uridine transporter proteins SLC29A1 and SLC29A2 are abundantly expressed in HEK293 and HeLa cell lines, thus 4SU is rapidly incorporated into newly transcribed RNA [100,101,102]. With the increasing need, the enrichment-based and low-resolution sequencing methods are not satisfying and single-base resolution is highly necessary. Although 4SU has been proven to generate a low level of T-to-C mutation in RNA sequencing [103], this information of mutation rate cannot be reliably used to identify new transcripts. The development of 4SU-based bioorthogonal chemical reactions to achieve high base mutation rate in RNA sequencing is highly rewarding.

In recent years, several chemical sequencing methods for 4SU detection have been developed. In 2017, Herzog et al. reported the method of thiol-linked alkylation for the metabolic sequencing of RNA (SLAM-seq) [104] by chemically modifying 4SU with iodoacetamide (IAA)-mediated alkylation to induce T-to-C mutation in RNA sequencing (Figure 3a). In this reaction, the thione of 4SU functions as a nucleophile, and IAA is covalently attached to 4SU via nucleophilic substitution (**24**), resulting in a reaction yield of greater than 98% within 15 min under optimal conditions. The 4SU alone contributes 10–11% T-to-C mutation, whereas IAA treatment increased the proportion by 8.5-fold to achieve about 94%. This strategy has also been extended to newly transcribed RNA sequencing in single cell [105,106]. In 2018, Schofield et al. developed the TimeLapse-seq [107] using an oxidative and nucleophilic aromatic substitution reaction with sodium periodate (NaIO${}_{4}$) and 2,2,2-trifluoroethylamine (TFEA) to convert 4SU to trifluoroethylated cytidine (**25**) which leads to T-to-C mutation in RNA sequencing. TimeLapse-seq has also been applied in single-cell profiling and has substantially outperformed SLAM-seq [108].

Another method, named thiouridine-to-cytidine sequencing (TUC-seq) [109,110], oxidizes 4SU to C through osmium tetroxide (OsO${}_{4}$) in ammonium chloride (NH${}_{4}$Cl) buffer, and enables 98% conversion of 4SU to C in 4 h without RNA degradation. Interestingly, the chemical treatment leads to nucleoside recoding to canonical nucleobase, which differs from the abovementioned modification adducts with chemical handles. In principle, TUC-seq can eliminate the effect or bias caused by chemical handles in RNA sequencing, and might be more accurate. However, OsO${}_{4}$ is more dangerous and unstable in solution and much harsher for RNA degradation, which may increase the difficulty and cost of the experiment. Chen et al. developed acrylonitrile-mediated uridine-to-cytidine conversion sequencing (AMUC-seq) [111] by using the Michael nucleophilic addition reaction of 4SU with acrylonitrile. The product S-cyanoethylated 4-thiouridine (Ce4SU,**26**) no longer pairs with A but with G, resulting in T-to-C mutations in RNA sequencing. More than 90% of 4SU can be converted to Ce4SU, however, the chemical treatment lasts for 4–10 h, which is longer than those of SLAM-seq and TimeLapse-seq, compromising the RNA substrate’s integrity.

The comparison of different sequencing methods has always been a tough issue because the associated experiments have not been performed under the same conditions and have their own systematic analysis of RNA kinetics. For this reason, Boileau et al. constructed a set of libraries using four methods (including 4SU-biotin conjugation-based enrichment method, SLAM-seq, TimeLapse-seq, and TUC-seq) and estimated the RNA decay rates using two different computational workflows [112]. The study found that the four methods are reliable and have comparable 4SU transformation efficiencies, and that the decay rates calculated by the two distinct computational methods are consistent across more than 11,600 human genes. However, after comparing protocols’ efficiency, reproducibility, and reliability, none can be considered the gold standard. It is worth mentioning that these chemical sequencing methods do not need enrichment. In general, the enrichment step may introduce a labeling bias, and additional experimental and/or computational processing are required to normalize the data [113,114].

### 3.2. 6SG for RNA Labeling and Detection

4SU has been well used in studying cellular RNA dynamics, but the synthesis and degradation of RNA cannot be accurately distinguished when cells are labeled with a single modified nucleoside in a pulse-chase manner. 4SU is also not ideal for studying the turnover of uridine-tailed RNAs, pseudouridylated RNAs and uridine-poor RNAs [115,116]. The development of new nucleoside analogs with a chemical sequencing potential will not only overcome these drawbacks, but also allow the dual-labeling applications. To meet the need, 6SG has been added to the toolkit.

TimeLapse-seq can be extended to recode 6SG into 2-aminoadenosine analog (**27**) to induce specific G-to-A mutations in RNA sequencing [117]. This method has been applied to determine the transcriptome RNA half-lives in K562 cells, and a positive correlation between 4SU-labeled and 6SG-labeled TimeLapse-seq has been obtained, indicative of the effectiveness of 6SG labeling. The TimeLapse chemistry was also applied in the Transcript Regulation Identified by Labeling with Nucleoside Analogues in Cell Culture (TILAC) method developed by Courvan et al. in 2022 [118]. TILAC used 4SU and 6SG as distinct metabolic markers to differentiate two RNA samples. It presents a novel technique to fairly compare RNA levels between different samples and to understand global changes in RNA levels, as well as specifically regulated transcripts.

Similarly, TUC-seq has been upgraded to TUC-seq DUAL method [119], which first uses NH${}_{4}$Cl and OsO${}_{4}$ to oxidize 6SG to 6-sulfoguanosine (6soG), and then converts 6soG to 6-hydrazino-2-aminopurine (**28**) in a buffered hydrazine solution. The thermodynamic stability of (**28**)-U pair is comparable to that of the natural A-U pair in short RNA oligonucleotides, but neither the pair of U-6SG nor U-6soG is stable [120]. In addition, the primer extension experiment using Superscript III reverse transcriptase revealed that 6SG and (**28**) had a moderate blocking effect on RT and were recognized as G and A, respectively, but 6soG had a high blocking rate and was recognized as G. The cellular 6SG/4SU dual labeling experiments demonstrated the feasibility of TUC-seq DUAL.

### 3.3. a${}^{6}$A for RNA Labeling and Detection

Shu et al. recently developed a${}^{6}$A-seq [43] that utilizes a${}^{6}$A analog to metabolically label cellular mRNAs and quantify them in an IP-free and mutation-based manner based on the iodination-induced cyclization chemistry mentioned above. After incubating Hela cells with a${}^{6}$A for a certain time, cellular mRNAs can be successfully labeled with a${}^{6}$A and can be identified by A-to-C/T mutation signals after treatment of iodination and subsequent RNA RT and high-throughput sequencing (Figure 3a). a${}^{6}$A-seq effectively characterizes the transcriptome-wide mRNA expression changes by calculating the global mutation reads, and distinguishes newly synthesized a${}^{6}$A-labeled mRNA from existing mRNA.

With the introduction of a${}^{6}$A, the tools available for studying RNA dynamics have expanded beyond 4SU and 6SG. There is still significant potential for the development of chemical sequencing tags based on cytosine. We anticipate the emergence of more diverse modified nucleosides as metabolic markers and more versatile chemical sequencing methods, which will greatly aid in gaining a deeper understanding of the intricate RNA dynamics.

### 3.4. aza${}^{5}$C for m${}^{5}$C Detection

In addition to harnessing the pathway of RNA post-modification, the random incorporation of artificial nucleosides into nascent RNA has also been successfully applied to detect natural nucleic acid modification sites. Khoddami et al. developed 5-azacytidine (aza${}^{5}$C, a cytidine analog)-mediated RNA IP (Aza-IP) [121] to specifically map the targets of RNA m${}^{5}$C MTases. The RNA MTases can react with aza${}^{5}$C in RNA to form a covalent product (**29**) through a thiol addition to the aza${}^{5}$C ring, leading to the disruption of RNA m${}^{5}$C methylation pathway (Figure 3b). Because of the presence of electronegative atoms surrounding C6 in (**29**), the N1–C6 bond is destabilized and then hydrolyzed to induce the formation of ring-opening product (**30**), which results in C to G mutation in RNA sequencing. On the basis of above principle, m${}^{5}$C can be identified at base resolution. Compared with RBS-seq, Aza-IP is bisulfite-independent and can enrich m${}^{5}$C sites. In general, overexpression of m${}^{5}$C RNA MTase is required in Aza-IP, which limits its applicability to some extent.

### 3.5. 5FU for DHU Detection

Individual-nucleotide resolution UV cross-linking and immunoprecipitation (iCLIP) [122] is widely used for studying RNA-protein interactions. The chemically stable RNA-protein conjugate generally results in cDNA truncation rather than mutation during RT. Here, an example is introduced, showing that metabolic labeling of a nucleoside on RNA induces chemical crosslinking between RNA and RNA-modifying enzyme and subsequent RT truncation.

DHU is one of the most conserved and abundant modified bases in tRNA. Recent evidences suggest that the distribution of DHU in the transcriptome is broader than expected (Figure 3c) [123]. Dai et al. developed a chemo-proteomic strategy to map DUS3L (a DHU synthase homolog)-dependent DHU modifications across the transcriptome [124]. The working principle given by Hamdane and colleagues is as follows [125]. First, nicotinamide adenine dinucleotide phosphate (NADPH) reduces flavin mononucleotide (FMN) to FMNH${}^{-}$. Then, FMNH${}^{-}$ transfers its hydride to C6 of 5FU to form adduct (**31**). DUS3L’s cysteine protonate C5 and forms 5-fluorodihydrouridine (**32**). Last, the formation of the covalent bond between cysteine and (**32**) by nucleophilic substitution of fluorine results in a covalent RNA-enzyme conjugate (**33**), which can cause cDNA truncation during RT. The metabolic labeling with 5-fluorouridine (5FU) validated the above strategy. The 5FU-iCLIP identified DHU sites to be U46-48 positions on 28s tRNA and also revealed a small number of DUS3L crosslinking peaks in mature mRNA. However, additional orthogonal validation is required to determine the presence of DHU on mammalian mRNA.

## 4. Conclusions and Outlook

In this work, we review the current state-of-the-art chemical sequencing methods for detecting both cellular natural RNA chemical modifications and metabolically installed tags on RNA. These technologies have significantly advanced the emerging field of RNA modifications and have prompted our understanding on sophisticated RNA metabolism and functions. Yet some issues remain. First, there are still few RNA modifications that can be detected using the chemical sequencing approach. It is rewarding to develop methods to locate other RNA modifications or discover new modifications. Second, the reliable RNA detection methods for rare and precious samples are lacking. Especially, a robust method for single cell analysis is not available. Third, simultaneous profiling of multiple modifications awaits exploration. ioorthogonal chemical/enzymatic reactions capable of carrying out diverse kinds of bases recoding are required, and are expected to possess higher reaction efficiency and sensitivity. Fourth, the modification stoichiometry should be carefully characterized because it is closely associated with the cellular functions. Fifth, the nucleoside analogs with chemical sequencing power are very rare. If we simultaneously label RNA transcripts in various kinds of cells, different chemical tags are needed. At current, 4SU, 6SG, and a${}^{6}$A are available chemical tags to metabolically replace U, G, and A in RNA, respectively, however, an effective cytidine analog with chemical sequencing power remains to be developed. Last, simplicity, low cost, and a standardized data analysis protocol are highly necessary in the sequencing method.

It should be highlighted that chemical sequencing methods have helped researchers elucidate the mechanism regarding how nucleic acid modification regulates gene expression. The future prospect of chemical sequencing will not only be limited to cellular system, but also be extended to disease diagnosis via the detection of RNA modifications in body fluids. It should be envisioned that the further developments will lead to broader clinical applications and benefit human health.

## Data Availability

Not applicable.
